# Effect of systemic arterial hypertension and use of antiproteinuric drug in induction therapy for lupus nephritis

**DOI:** 10.31744/einstein_journal/2020AO5322

**Published:** 2020-08-28

**Authors:** Eduardo Grecco Matta, Danielle Arraes Rubini, Nafice Costa Araújo

**Affiliations:** 1 Hospital do Servidor Público Estadual São PauloSP Brazil Hospital do Servidor Público Estadual, São Paulo, SP, Brazil.

**Keywords:** Lupus nephritis, Proteinuria, Renal insufficiency, chronic, Hypertension, Renin-angiotensin system

## Abstract

**Objective:**

To evaluate the therapeutic response to induction treatment in lupus nephritis patients.

**Methods:**

A total of 29 patients diagnosed with systemic lupus erythematosus and biopsy-proven nephritis were divided into two groups, one with hypertensive individuals and another non-hypertensive patients. The hypertensive patients included were on drugs with antiproteinuric effect. The induction treatment comprised mycophenolate mofetil or cyclophosphamide, based on 24-hour proteinuria and serum creatinine parameters for therapeutic evaluation after 6 months of intervention. The retrospective evaluation of the follow-up was made based on information collected from the medical records.

**Results:**

Patients with and without hypertension presented similar behaviors of proteinuria (p=0.127) and creatinine (p=0.514) over time. For proteinuria, only the time effect (p=0.007), but not hypertensive effect (p=0.232), was found. There was a reduction in proteinuria levels (reduction by 3.28g/24 hours, on average) from the beginning to the final measurement. As to creatinine, no hypertensive (p=0.757) or time (p=0.154) effects were found.

**Conclusion:**

Similarity in behavior of proteinuria was observed, after induction treatment for nephritis, taking into account the hypertensive effect. The prior condition did not hinder these patients reaching the recommended proteinuria goal.

## INTRODUCTION

In systemic lupus erythematous (SLE), kidney involvement has extreme impact on survival and quality of life of patients.^(1,2)^

Most patients with lupus nephritis (LN) have immune complex-mediated glomerular disease, often associated with tubulointerstitial changes. Renal vasculature involvement is common, ranging from vascular immune deposits to fibrinoid necrosis and thrombotic microangiopathy.^(2)^

Cardiovascular diseases are the main causes of death in these patients, however, due to systemic involvement and treatment with immunosuppressants, infectious diseases and renal dysfunction stand out as important causes of death.^(3)^

The importance of renal involvement is evident since approximately 10% to 30% of individuals with LN progress to established chronic kidney disease (CKD), requiring renal replacement therapy, which leads to increased morbidity and mortality.^(4)^

The role of kidney biopsy is therefore essential, since clinical, immunological or laboratory parameters do not predict histological findings. Biopsy helps defining the mechanism of kidney involvement, guiding the treatment.^(5)^

According to the European League Against Rheumatism (EULAR) and the American College of Rheumatology (ACR), it is recommended that kidney biopsy be carried out whenever there is a sign of renal involvement, especially proteinuria ≥0.5g/24 hours with glomerular dysmorphic hematuria and/or casts.^6^

The severity of this disease varies, depending on the location of the immune complex deposit and quality of autoantibodies. Some forms do not require kidney-targeted therapy. Most have good long-term results, without the risks related to exposure to an immunosuppressive regimen.^(5,8,9)^

The treatment of LN is an emergency among the proliferative forms, considering the risk of progression to CKD.^(4)^ Lupus nephritis is initially treated with steroids, used in conjunction with other immunosuppressants in induction therapy, such as mycophenolate mofetil (MMF) and cyclophosphamide (CP). Calcineurin inhibitors or rituximab are recommended as complementary alternative options in LN.^(4,10)^

The basis of treatment includes anti-inflammatory and immunosuppressive agents to interrupt autoimmune chains. The treatment induction phase includes an interval of 3 to 6 months, followed by the maintenance phase. Determining the end of treatment is not well established.^(1)^ Such therapeutic regimens showed a rise of approximately 80% in five-year survival, but the rates of complete kidney response in one year are only 10%-40%.^(1)^

Proteinuria and serum creatinine levels have been widely accepted as short-term response measurements, since they are non-invasive and accessible, reflecting the severity of kidney damage.^(11)^

The short-term renal response parameters, using long-term data obtained from the Euro-Lupus Nephritis study, demonstrated an absolute proteinuria level of 0.8g per day, at 12 months after randomization, is the best individual predictor of good renal prognosis over a period of 7 years.^(11)^

Significant percentage of patients with LN progress to CKD, although this is not a significant cause of the disease. Systemic Arterial Hypertension (SAH) and *diabetes mellitus* (DM) are the most important morbidities associated with the development of kidney dysfunction. *Diabetes mellitus* is the leading cause of CKD in developed countries and is close to the figures of hypertension and chronic glomerulonephritis as the main causes in developing countries.^(4,12-14)^ In the case of SAH, the pathogenesis is not well known; it is likely the result of many genetic and environmental factors that have multiple composite effects on cardiovascular and renal structures and functions.^(15)^

The roles of the immune system and chronic inflammation in the development of hypertension and its complications are increasingly well established. Understanding the development of hypertensive disease in an inflammatory setting may have great clinical relevance in establishing the relation between autoimmune disorder and the progression of increased peripheral vascular resistance.^(16)^

Disorders generated by changes in the renal microvasculature associated with the deposition of immune complexes play a central role in the development of CKD and, possibly, have a direct effect on the therapeutic response to nephritis.^(12,16)^

## OBJECTIVE

To comparatively evaluate the response to induction therapy for lupus nephritis, considering proteinuria and serum creatinine levels in patients diagnosed as systemic arterial hypertension, on antiproteinuric medication, and patients not presenting this disease and not using this medication.

## METHODS

A retrospective study of patients with biopsy-proven LN, followed from January 2006 to February 2018, at a rheumatology outpatient clinic in the city of São Paulo (State of São Paulo - SP).

This study included 29 patients, of both sexes and all ages. Systemic arterial hypertension was used as variable. The primary endpoint was to study the response to therapy in LN patients with SAH as compared to individuals with no diagnosis of hypertension, treated with CP or MMF induction, with absolute proteinuria levels as the response parameter. The secondary endpoint included changes in serum creatinine levels and the percentage decrease in proteinuria.

The studied population consisted of patients diagnosed with SLE, according to the ACR 1997 criteria, and the Systemic Lupus International Collaborating Clinics (SLICC) from 2012.^(7,17)^ Lupus nephritis was diagnosed based on the findings of the biopsy, according to the classification of the International Society of Nephrology (ISN)/Renal Pathology Society (RPS).^(5,7,8)^

All biopsy-proven cases were included in our study. Patients of both sexes and different age groups were considered. Among the group of patients with SAH, only those who, at the time of disease activity were already on antihypertensive drugs with antiproteinuric effect - angiotensin-converting enzyme inhibitors (ACEi) or angiotensin receptor blockers (ARB), were included.

Patients with the following conditions were excluded from the study: CKD stage IV or more advanced; DM patients; chronic glucocorticoid users; previous treatment with CP or MMF for less than a year from the last induction; use of medication other than MMF and CP as induction therapy.

For the purpose of analyzing the response to treatment, we established the criteria proposed by the consensus of the Brazilian Society of Rheumatology. Complete remission (CR) was defined as proteinuria <0.5g in 24 hours, and partial remission (PR) was defined as >50% reduction in initial proteinuria with value <3.0g in 24 hours.^(5)^

The selected patients were submitted to therapy with CP or MMF. Induction was conducted including pulse therapy with methylprednisolone (0.5 to 1g intravenously, or 10 to 30mg/kg/ day, for 3 consecutive days). Prednisone doses remained between 0.5 to 1mg/kg/day, for 3 to 4 weeks, with subsequent tapering and aiming at a dose of 5 to 10mg/day, in 6 months. In conjunction with steroid therapy, intravenous CP at 0.5 to 1g/m^2^ of body surface area was included monthly, for 6 months, or 0.5g intravenous CP, every 15 days for 3 months, or MMF 2 to 3g/day.

These patients were selected based on the criteria described, and considering the purpose of assessing the impact of SAH in the induction therapy, they were divided into two groups: Group 1, nine patients with no diagnosis of SAH; and Group 2, 20 subjects with diagnosis of SAH.

The retrospective evaluation of the follow-up was done based on information collected from medical records, selecting the last laboratory data prior to the beginning of induction therapy and, in the evaluation of the therapeutic response, data from 3 to 6 months from the beginning of treatment were selected. The 24-hour urinary protein was measured as gram/24 hours, and serum creatinine as mg/dL.

### Statistical methods

First, data were descriptively analyzed. For categorical variables, absolute and relative frequencies were presented and, for numerical variables, summary measures.

Due to the sample size, the existence of associations between two categorical variables was verified using Fisher’s exact test. The comparison of mean age by SAH was done by Student’s *t* test. The data distribution normality was verified using the Kolmogorov-Smirnov test. Variations in the percentages of change and in relative proteinuria were verified via Student’s *t* test for one sample.

In order to assess the behavior of proteinuria and creatinine at data points and SAH, the generalized estimating equation (GEE) models were used with identity link function and normal marginal distribution.

For all statistical tests, a significance level of 5% was set. The GEE models were estimated using STATA 12. For other analyses, the software (SPSS) 20.0 was used.

## RESULTS

The data of 29 patients were evaluated, the mean age was 50.1 years, range 23-78 years, and 93.1% were female.

When comparing the biopsy results between Groups 1 and 2, there was predominance of proliferative glomerulonephritis (class III/IV), with 55.5% and 70%, followed by class V, with 33.3% and 25%, respectively; however, there were no distinct distributions in relation to biopsies (p=0.393). On the other hand, there were differences in the mean age (p=0.049), which was lower in Group 1.

The evaluation of proteinuria and creatinine behavior at data points and SAH was done using the GEE model.

As depicted in table 1, Groups 1 and 2 presented similar behaviors of proteinuria (p=0.127) and creatinine (p=0.514) over time.

**Table 1:** Results of the generalized estimating equations model for proteinuria and creatinine

Variables	Coefficient (95%CI)	p value
Proteinuria, g/24 hours		
SAH*	-1.70 (-4.49-1.09)	0.232
Time, post^†^	-3.28 (-5.68- -0.88)	0.007
Interaction time *versus* SAH	1.99 (-0.56-4.54)	0.127
Constant	4.35 (1.76-6.94)	0.001
Creatinine, mg/dL		
SAH*	0.09 (-0.49-0.68)	0.757
Time^†^	-0.29 (-0.69-0.11)	0.154
Interaction time tempo *versus* SAH	0.15 (-0.30-0.61)	0.514
Constant	1.10 (0.76-1.44)	<0.001

n=58 observations concerning 29 patients.

* no SAH as reference; ^†^ using pre induction therapy values as reference.

95% CI: 95% confidence interval; SAH: systemic arterial hypertension.

**Figure 1:**
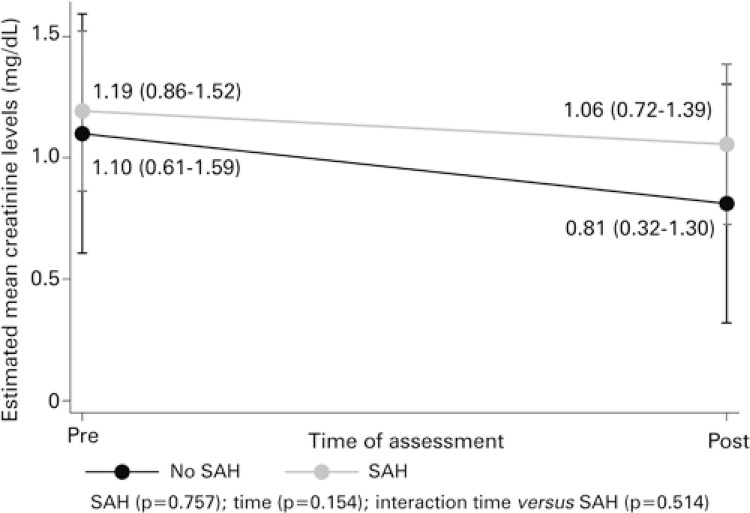
Estimation of the means for creatinine in evaluation point, as per hypertension SAH: systemic arterial hypertension.

**Figure 2:**
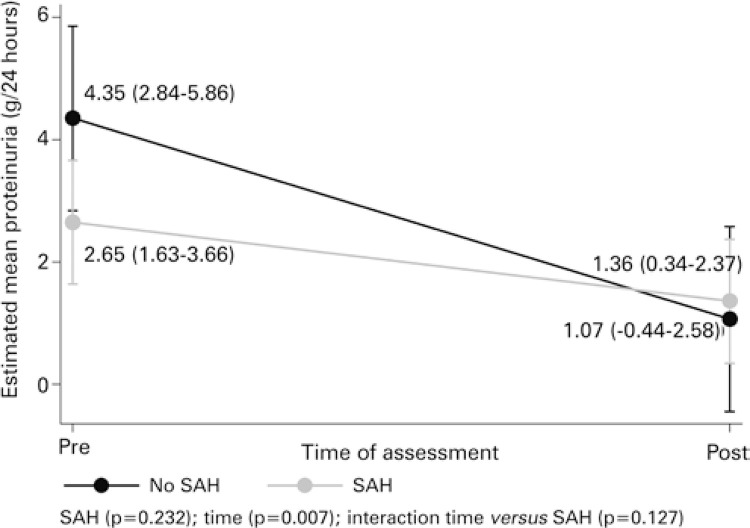
Estimation of the means for urinary protein in evaluation point, as per systemic arterial hypertension SAH: systemic arterial hypertension.

In addition, for proteinuria, only the effect of time was seen (p=0.007), not of SAH (p=0.232). Therefore, a reduction in urinary protein levels is noted (reduction by 3.28g/24hours, on average) from the beginning to the post-induction moment, as shown in table 2. For creatinine, no effects were found of SAH or time (p=0.757 and p=0.154, respectively). These average behaviors are shown in figures 1 and 2.

**Table 2:** Summary-measurements of urinary protein and creatinine as per systemic arterial hypertension

Variables	Assessment data point		
	Pre	After 3-6 months	Variation*
Urinary protein, g/24 hours			
No SAH			
Mean±SD	4.35±4.13	1.07±1.08	-3.28±3.82
Median (Min-Max)	3.2 (1.00-14.79)	0.37 (0.15-3.22)	-2.26 (-13.18- -0.79)
SAH			
Mean±SD	2.65±2.38	1.36±1.73	-1.29±2.01
Median (Min-Max)	2.26 (0.46-9.80)	0.57 (0.12-6.85)	-1.35 (-6.01-3.46)
Creatinine, mg/dL			
No SAH			
Mean±SD	1.10±0.54	0.81±0.15	-0.29±0.63
Median (Min-Max)	0.90 (0.70-2.30)	0.80 (0.50-1.00)	0.00 (-1.80-0.10)
SAH			
Mean±SD	1.19±1.09	1.06±0.64	-0.14±0.51
Median (Min-Max)	0.90 (0.60-5.70)	0.90 (0.60-3.60)	0.00 (-2.10-0.40)

n=9 and n=20, respectively for Groups 1 and 2.

^*^ post - pre-induction therapy values. SAH: systemic arterial hypertension; SD: standard deviation.

Since the groups had different mean ages, the regression model was adjusted including age in the model.

As depicted in table 3, age was significant for proteinuria (p=0.019), but not for creatinine (p=0.745). Therefore, for proteinuria, a reduction by 0.06g/24 hours was seen for each increase by one year of age. Again, it can be noted that patients with SAH and without SAH presented similar behaviors of proteinuria (p=0.055) and creatinine (p=0.478) over time. In addition, for proteinuria, only the effect of time (p<0.001) was found, not of SAH (p=0.255). There is therefore a reduction in proteinuria levels (reduction by 3.28g/24 hours, on average) from the beginning to the post induction moment. For creatinine, there were no effects of SAH (p=0.690) or time (p=0.104).

**Table 3:** Results of the model generalized estimation equations for proteinuria and creatinine with adjustment for the inclusion of age in the model

Variables	Coefficient (CI95%)	p value
Proteinuria		
SAH*	-1.05 (-2.85-0.76)	0.255
Time, post^†^	-3.28 (-4.97- -1.59)	<0.001
Interaction time *versus* SAH	1.99 (-0.05-4.02)	0.055
Age	-0.06 (-0.12--0.01)	0.019
Constant	7.15 (4.40-9.90)	<0.001
Creatinine		
SAH*	0.13 (-0.50-0.76)	0.690
Time, post^†^	-0.29 (-0.64-0.06)	0.104
Interaction time *versus* SAH	0.15 (-0.27-0.57)	0.478
Age	0.00 (-0.03-0.02)	0.745
Constant	1.26 (0.20-2.31)	0.020

n=58 observations concerning 29 patients.

* no SAH as reference; ^†^ using the pre-induction therapy values as reference.

95%CI: 95% confidence interval; SAH: systemic arterial hypertension.

**Figure 3:**
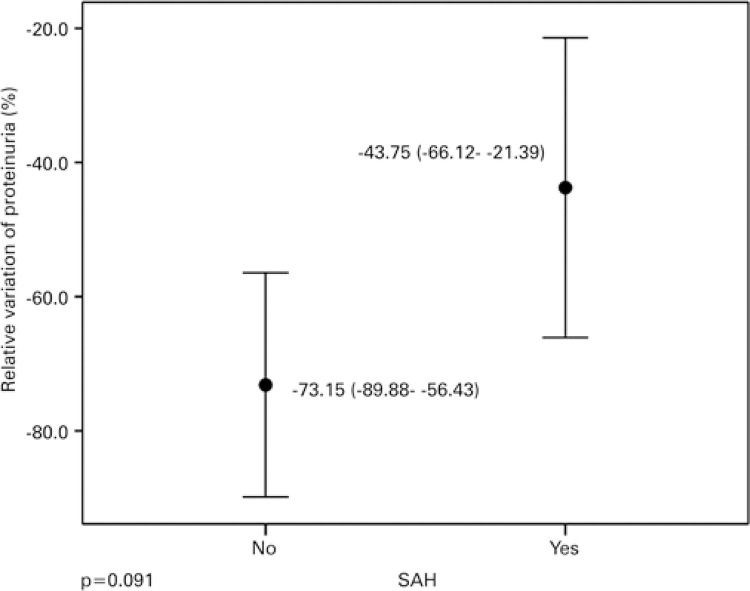
Proportion of proteinuria change after the intervention and respective 95% confidence interval, as per systemic arterial hypertension SAH: systemic arterial hypertension.

**Figure 4:**
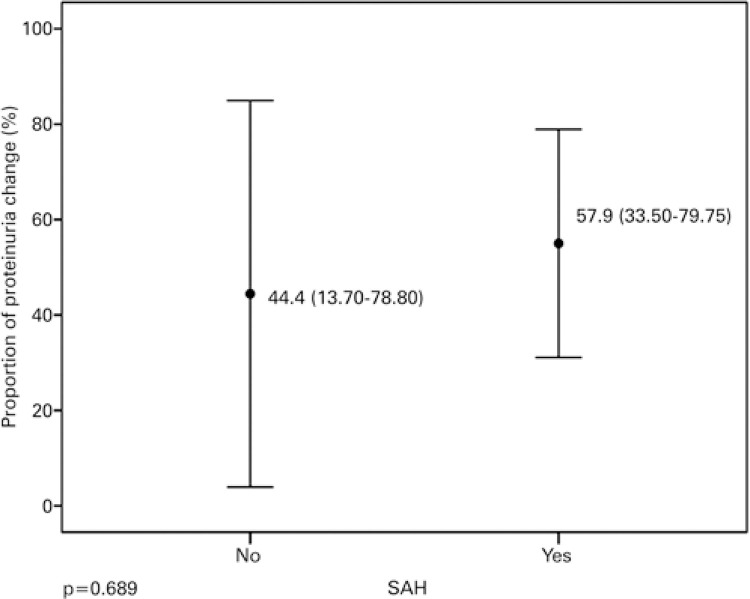
Mean relative proteinuria variation and respective 95% confidence interval, as per systemic arterial hypertension SAH: systemic arterial hypertension.

There was a reduction in the percentage of change in both Groups 1 (p=0.013) and 2 (p=0.001), as demonstrated in figure 3, which shows a greater variation in Group 1. There was also a relative reduction in proteinuria in patients in Group 1 (p<0.001) and Group 2 (p=0.001), graphically represented in figure 4. As depicted in table 4, there were no distinct distributions of changes in proteinuria consequent to SAH (p=0.689) or differences in means of relative variations in proteinuria consequent to SAH (p=0.091).

**Table 4:** Distribution of patients by classification of post-induction proteinuria and relative variation of proteinuria

Variables	Total	No SAH	SAH	p value
Proteinuria* post				0.689
No change (≤0.5g/24 hours)	13/28 (46.4)	5/9 (55.6)	8/19 (42.1)	
Altered (>0.5g/24 hours)	15/28 (53.6)	4/9 (44.4)	11/19 (57.9)	
Relative variation^†^ of proteinuria				0.091^‡^
Mean±SD	-52.88±43.32	-73.15±21.76	-43.75±47.79	
Median (Min-Max)	-57.24 (-96.00; 102.06)	-79.00 (-96.00; -38.67)	-52.76 (-92.22; 102.06)	

Results expressed as n (%) or %.

Relative variation of proteinuria: n=9 and n=20, respectively for Groups 1 and 2. Kolmogorov-Smirnov test for distribution normality of relative variation of proteinuria (p=0.317).

* only for patients with abnormal proteinuria on the initial assessment; ^†^ post- and pre-induction therapy values. ^‡^ Fisher’s exact test or Student *t* test.

SAH: systemic arterial hypertension; SD: standard deviation.

## DISCUSSION

Based on analysis of the results and taking into account the data obtained in the primary endpoint, there was similarity in the behavior of proteinuria among patients with and without SAH. Both obtained an average reduction by 3.28g/24 hours. This result suggests the fact that hypertensive patients already on antiproteinuric drug and, possibly, presenting SAH-related microvasculature lesions, does not have a significant direct effect on this parameter.

Considering also the elements of the primary endpoint, it is evident that the serum creatinine level, as well as urinary protein, suffer no secondary effect of the variable SAH. What is noted in the graphic analysis is higher serum creatinine levels among hypertensives, which we attribute to age and SAH.

In progressive nephropathies, such as hypertensive nephropathy, severe dysfunction of the glomerular capillary barrier of circulating proteins causes protein overload on tubular epithelial cells, and activation of the intrarenal complement, which is responsible for the propagation of damage to the tubular-interstitial compartment.^(18)^

The abnormal passage of plasma proteins through the glomerular capillary wall is responsible for more podocyte lesions and progression to glomerulosclerosis. However, we must highlight other mechanisms that lead to the activation of proximal tubular cells, interstitial inflammation and fibrosis, such as albumin toxicity, in addition to transferrin and ultra-filtered immunoglobulins, and activation of the complement pathway.^(18)^

One of the first clinical trials supporting the concept of proteinuria as an independent risk factor for the progression of kidney disease was the Modification of Diet in Renal Disease (MDRD). Since then, numerous analyzes have confirmed this observation. Consequently, hypotensive medications for patients with kidney disease are based on efficacy of these agents in reducing proteinuria.^(19)^

Several studies that demonstrated renoprotection with ACE inhibitors or ARB also reported reduction in proteinuria. An analysis of studies in patients with hypertension and diabetic nephropathy, and in non-diabetic patients with hypertension and nephropathy showed the initial changes in proteinuria had a favorable relation with severity of long-term kidney deterioration.^(19)^

Renal parameters of proteinuria and serum creatinine have been widely accepted as short-term response measurements, since they are non-invasive, easily quantifiable and can reflect kidney injury. They are used individually and in combination in clinical trials to determine the efficacy of new drugs in lupus patients, although their usefulness in predicting long-term renal outcome has not been studied.^(12)^

Dall’Era et al.,^(11)^ demonstrated results that provide a convincing argument for the use of the absolute level of proteinuria alone, as a measurement of therapeutic response and a valuable long-term prognostic marker. However, the absolute serum creatinine level after one year did not add much to the predictive value of the absolute level of proteinuria alone. In this study, they also suggested the frequently used criterion of 0.5g of proteinuria per day may not be the best.^(12)^

A study in the Brazilian population showed proteinuria less than 0.8g/24 hours at 12-month follow-up was the single best predictor of a good long-term kidney outcome, in an ethnically mixed group of patients with severe nephritis. Regarding histological classes, membranous glomerulonephritis appears to have a more favorable long-term course as compared to the proliferative forms of the disease, and there are some concerns about the performance of the proposed proteinuria target, which may be different in patients with proliferative forms as compared to membranous disease.^(20)^

We expected to find in our study that hypertensive patients, due to the fact they were already on a nephroprotective agent, would present a more satisfactory response in relation to non-hypertensive patients in the short run, what was not confirmed.

During the analysis of the secondary endpoint, we evaluated that, although patients with hypertension had a lower initial proteinuria value, the percentage decrease in proteinuria of non-hypertensive patients did not show a comparative statistical difference related to the two groups, despite a reduction of 73.15% between those without SAH *versus* 43.75% of those with SAH.

Such a difference in decrease is expected, since hypertensive patients who had previously used an antiproteinuric drug already had an initial value lower than the others. However, another factor to be considered is the likely residual albuminuria related to hypertensive disease and not necessarily the activity of lupus disease.^(21)^

With regard to the targeted proteinuria, 55.6% of non-hypertensive subjects reached the established target of 0.5g/24 hours compared to 42.1% of hypertensive subjects. These data demonstrated that 13.5% more non-hypertensive patients submitted only to induction therapy reached the target value. This difference was not significant in our analysis, and regarding this result, we must take into account proteinuria secondary to underlying hypertensive disease.

The ideal target for proteinuria is debatable, nevertheless the importance of reaching it is unquestionable, since it is a marker of atherosclerosis and vascular disease in the general population and in patients with DM, with an estimated 50% higher risk of coronary atherosclerotic disease than healthy controls.^(22)^

Proteinuria from active lupus kidney disease or CKD in SLE is therefore associated with cardiovascular diseases and, hence, considered a prognostic factor. In patients with SLE, there is an increase in the mortality rate related to premature atherosclerosis, with a higher prevalence of significant coronary artery obstruction as compared to healthy controls. In general, patients with SLE are at higher risk for accelerated atherosclerosis than patients with DM.^(22)^

With the objective of increasing the survival of lupus patients with renal dysfunction, the proteinuria parameter appears as both diagnostic and key prognostic value for the follow-up of these individuals. Taking into account comorbidities associated with autoimmune diseases, such as hypertension and diabetes, greater care is needed; it is possible that these patients need individual proteinuria parameters to define disease activity or remission.^(22)^

Our study has an important limitation with regard to the samples size, in addition to their heterogeneity, hindering the statistical analysis. Nonetheless, since it includes only patients with biopsy, a considerable number of patients were excluded, making it difficult to find an adequate and homogeneous number for the groups.

## CONCLUSION

In this study, the similarity in the behavior of proteinuria is evident, after induction therapy for lupus nephritis, considering the effect of primary hypertension. Despite the difference in initial proteinuria between groups, after therapy there was no significant effect of hypertension on the percentage decrease in proteinuria. Therefore, previous comorbidity was not an obstacle for these patients to reach the recommended proteinuria goal.

From the results obtained, the need for in-depth studies with a larger number of patients is evident, in such a way that there is no greater interference regarding the heterogeneity between the samples.
