# Supplementary material for: Effect of systemic arterial hypertension and use of antiproteinuric drug in induction therapy for lupus nephritis
Source: Einstein (Sao Paulo). 2020 Aug 28;18:eAO5322. doi: 10.31744/einstein_journal/2020AO5322 (PMC7453831; doi:10.31744/einstein_journal/2020AO5322)
Supplement: Translation

# Translation: Efeito da hipertensão arterial sistêmica com uso de antiproteinúrico na terapia de indução da nefrite lúpica

**Authors:** Eduardo Grecco Matta, Danielle Arraes Rubini, Nafice Costa Araújo

**Affiliations:** 1 Brasil Hospital do Servidor Público Estadual, São Paulo, SP, Brasil.

**Keywords:** Nefrite lúpica, Proteinúria, Insuficiência renal crônica, Hipertensão, Sistema renina-angiotensina

## Abstract

**Objetivo:**

Avaliar resposta terapêutica a tratamento de indução de pacientes com nefrite lúpica.

**Métodos:**

Foram divididos em dois grupos 29 pacientes com diagnóstico de lúpus eritematoso sistêmico e nefrite comprovada por biópsia, sendo um de portadores de hipertensão e outro sem essa comorbidade. Dentre os hipertensos, foram incluídos aqueles que faziam uso de antiproteinúrico. O tratamento de indução foi realizado com micofenolato de mofetila ou ciclofosfamida, sendo utilizados os parâmetros de proteinúria de 24 horas e creatinina sérica para avaliação terapêutica após 6 meses da intervenção. A avaliação retrospectiva do seguimento foi realizada a partir de informações coletadas de prontuário.

**Resultados:**

Pacientes com e sem hipertensão apresentaram comportamentos similares de proteinúria (p=0,127) e creatinina (p=0,514) ao longo do tempo. Para a proteinúria, observou-se apenas o efeito de tempo (p=0,007), mas não de hipertensão (p=0,232). Houve redução nos níveis da proteinúria (redução de 3,28g/24 horas, em média) do início para o momento pós. Já para a creatinina, não se observou efeito de hipertensão (p=0,757) e tampouco de tempo (p=0,154).

**Conclusão:**

Observamos similaridade no comportamento da proteinúria, após tratamento de indução para nefrite, levando em conta o efeito hipertensão. A comorbidade prévia não se mostrou impeditiva para que estes pacientes atingissem a meta de proteinúria preconizada.

## INTRODUÇÃO

No lúpus eritematoso sistêmico (LES), o acometimento renal é de extrema importância no impacto da sobrevida e da qualidade de vida dos pacientes.^(1,2)^

A maioria dos pacientes com nefrite lúpica (NL) apresenta doença glomerular mediada por imunocomplexos, frequentemente associada a alterações tubulointersticiais. O envolvimento da vasculatura renal é comum, variando de depósitos imunes vasculares à necrose fibrinoide e microangiopatia trombótica.^(2)^

As doenças cardiovasculares são as principais causas de morte nesses pacientes, porém, devido ao acometimento sistêmico e tratamento com imunossupressores, os quadros infecciosos e a disfunção renal se destacam como importantes causas de óbito.^(3)^

A importância do acometimento renal fica evidente à medida que cerca de 10% a 30% dos indivíduos com NL evoluem para doença renal crônica (DRC) estabelecida com necessidade de terapia de substituição renal, o que acarreta em aumento de morbimortalidade.^(4)^

Assim, o papel da biópsia renal é imprescindível, uma vez que os parâmetros clínicos, imunológicos e laboratoriais não predizem os achados histológicos. Sua realização auxilia na definição de mecanismo de acometimento renal, guiando o tratamento de forma específica.^(5)^

Segundo a *European League Against Rheumatism* (EULAR) e o *American College of Rheumatology* (ACR), é recomendado que a biópsia renal seja feita sempre que houver sinal de envolvimento renal, especialmente proteinúria ≥0,5g/24 horas com hematúria dismórfica glomerular e/ou cilindros celulares.^(6,7)^

A gravidade dessa enfermidade é variável, dependendo do local de depósito do imunocomplexo e da qualidade dos autoanticorpos. Algumas formas não necessitam de terapia direcionada ao rim. A maioria apresenta bons resultados em longo prazo, sem os riscos relacionados à exposição a um regime imunossupressor.^(5,8,9)^

Dentre as formas proliferativas, o tratamento da NL é uma urgência, tendo em vista o risco de progressão para DRC.^(4)^ A NL é inicialmente tratada com corticosteroide, usado em conjunto com outros agentes imunossupressores na terapia de indução − dentre eles o micofenolato de mofetila (MMF) e a ciclofosfamida (CFM). De forma complementar na NL, os inibidores da calcineurina ou do rituximabe são recomendados como opções alternativas.^(4,10)^

A base do tratamento envolve agente anti-inflamatório e imunossupressor, para interromper as cadeias autoimunes. A fase de indução do tratamento engloba um intervalo de 3 a 6 meses, seguida de fase de manutenção. A determinação de fim de tratamento não é bem estabelecida.^(1)^ Tais esquemas terapêuticos mostraram melhora em sobrevida para cerca de 80% em 5 anos, porém as taxas de resposta renal completa em um ano são apenas 10%-40%.^(1)^

A proteinúria e os níveis séricos de creatinina têm sido amplamente aceitos como medidas de resposta de curto prazo, pois são medidas não invasivas e acessíveis, que refletem o grau de lesão renal.^(11)^

Os parâmetros de resposta renal a curto prazo, usando dados de longo prazo obtidos do estudo *Euro-Lupus Nephritis*, demonstram que um nível absoluto de proteinúria de 0,8g ao dia, aos 12 meses pós-randomização, é o melhor preditor individual de bom prognóstico renal em um período de 7 anos.^(11)^

Percentual significativo dos pacientes com NL progride para quadro de DRC, porém esta não se apresenta como causa significativa da enfermidade. Dentre essas enfermidades, configuram-se a hipertensão arterial sistêmica (HAS) e o *diabetes mellitus* (DM) como principais morbidades associadas ao desenvolvimento de disfunção renal. O DM é a causa líder de DRC em países desenvolvidos e está próximo aos números de hipertensão e glomerulonefrite crônica como principais causas nos países em desenvolvimento.^(4,12-14)^ No caso da HAS, a patogênese é pouco conhecida, mas é provavelmente o resultado de numerosos fatores genéticos e ambientais que têm múltiplos efeitos compostos na estrutura e nas funções cardiovascular e renal.^(15)^

O papel do sistema imunológico e da inflamação crônica, no desenvolvimento da hipertensão, e suas complicações são cada vez mais bem estabelecidos. A compreensão do desenvolvimento da doença hipertensiva em um cenário inflamatório pode ter grande relevância clínica, a fim de contribuir para a relação entre a desordem autoimune e a progressão do aumento da resistência vascular periférica.^(16)^

As desordens geradas por alteração na microvasculatura renal associada à deposição de imunocomplexos desempenham papel central no desenvolvimento de DRC e, possivelmente, tenham efeito direto na resposta terapêutica à nefrite.^(12,16)^

## OBJETIVO

Avaliar comparativamente a resposta à terapia de indução de nefrite lúpica, levando em conta níveis de proteinúria e níveis séricos de creatinina, entre pacientes com diagnóstico de hipertensão arterial sistêmica em uso de medicação antiproteinúrica e pacientes sem essa enfermidade e sem uso dessa medicação.

## MÉTODOS

Estudo retrospectivo sobre pacientes com NL comprovada por biópsia, em acompanhamento de janeiro de 2006 a fevereiro de 2018, em um ambulatório de reumatologia, em São Paulo (SP).

Foram incluídos neste estudo 29 pacientes, de ambos os sexos e de todas as idades. Como variável, foi utilizada a presença de HAS. O desfecho primário foi estudar a resposta à terapia em pacientes com NL portadores de HAS em relação a indivíduos sem esse diagnóstico, tratados com indução, usando CFM ou MMF, tendo como parâmetro de resposta os níveis absolutos de proteinúria. O desfecho secundário inclui a alteração dos níveis de creatinina sérica e o percentual de decréscimo da proteinúria.

A população estudada foi constituída por pacientes diagnosticados com LES, de acordo com os critérios do ACR, de 1997, e do *Systemic Lupus International Collaborating Clinics* (SLICC), de 2012.^(7,17)^ A NL foi diagnosticada com base nos achados da biópsia, segundo a classificação da *International Society of Nephrology* (ISN)/*Renal Pathology Society* (RPS).^(5,7,8)^

Todos os casos comprovados por biópsia foram incluídos em nosso estudo. Pacientes de ambos os sexos e grupos etários variados foram considerados. Dentre o grupo de pacientes com HAS, foram incluídos apenas aqueles que, no momento da atividade da doença, já faziam uso de anti-hipertensivos com caráter antiproteinúrico, fossem inibidores da enzima conversora de angiotensina (IECA) ou bloqueadores de receptores de angiotensina (BRA).

Pacientes com as seguintes condições foram excluídos do estudo: DRC estágio IV ou acima; portadores de DM; usuários crônicos de glicocorticoides; tratamento prévio com CFM ou MMF a menos de um ano da última indução; e uso de outra medicação que não fosse MMF e CFM como terapia de indução.

Para fins de análise de resposta ao tratamento, estabelecemos critérios propostos pelo consenso da Sociedade Brasileira de Reumatologia. A remissão completa (RC) foi definida como proteinúria <0,5g em 24 horas, e a remissão parcial (RP) foi definida como redução >50% da proteinúria inicial com valor <3,0g por 24 horas.^(5)^

Os pacientes selecionados foram submetidos à terapêutica com CFM ou MMF. A indução foi realizada incluindo pulsoterapia com metilprednisolona (0,5 a 1g por via intravenosa, ou 10 a 30mg/kg/dia, durante 3 dias consecutivos). As doses de prednisona permaneciam entre 0,5 a 1mg/kg/dia, por 3 a 4 semanas, com redução subsequente e objetivo de alcançar a dose de 5 a 10mg/dia, em 6 meses. Em conjunto com a corticoterapia, foi incluído CFM por via intravenosa 0,5 a 1g/m^2^ de árae de superfície corpórea, mensalmente, durante 6 meses, ou CFM por via intravenosa 0,5g, a cada 15 dias, durante 3 meses, ou MMF 2 a 3g ao dia.

Esses pacientes foram selecionados a partir dos critérios descritos, e considerando a finalidade de avaliação da relação da HAS na terapia de indução, eles foram divididos em dois grupos: Grupo 1, composto por nove pacientes sem o diagnóstico de HAS; e Grupo 2, composto por 20 indivíduos com esse diagnóstico.

A avaliação retrospectiva do seguimento foi realizada a partir de informações coletadas de prontuário, sendo selecionados os últimos dados laboratoriais anteriores ao início da terapia de indução e, na avaliação da resposta terapêutica, foram selecionados dados de 3 a 6 meses do início do tratamento. Foram utilizados valores de proteinúria de 24 horas na unidade de grama por 24 horas e valores séricos creatinina em mg/dL.

### Análise estatística

Inicialmente, os dados foram analisados descritivamente. Para as variáveis categóricas, foram apresentadas frequências absolutas e relativas e, para as variáveis numéricas, medidas-resumo.

As existências de associações entre duas variáveis categóricas foram verificadas utilizando-se o teste de exato de Fisher, devido ao tamanho da amostra. A comparação de médias de idade por HAS foi realizada utilizando-se o teste *t* de Student. A normalidade na distribuição dos dados foi verificada empregando-se o teste de Kolmogorov-Smirnov. As variações das percentagens de alteração e relativa da proteinúria foram verificadas via teste *t* de Student para uma amostra.

Para se avaliar o comportamento da proteinúria e creatinina por momentos de avaliação e HAS, foram empregados os modelos de Equações de Estimativas Generalizadas (GEE) com função de ligação identidade e distribuição marginal normal.

Para todos os testes estatísticos, foi adotado nível de significância de 5%. Os modelos GEE foram estimados utilizando-se o STATA 12. Para as demais análises, empregou-se o software (SPSS) 20.0.

## RESULTADOS

Foram avaliadas as informações de 29 pacientes, cuja média das idades foi de 50,1 anos, sendo observadas idades mínima de 23 anos e máxima de 78 anos, e 93,1% eram do sexo feminino.

Ao serem comparados os resultados de biópsia entre os Grupos 1 e 2, nota-se a predominância de glomerulonefrite proliferativa (classe III/IV), com 55,5% e 70%, seguida da classe V, com 33,3% e 25%, respectivamente, porém não se verificaram distribuições distintas em relação às biópsias (p=0,393). Por outro lado, verificaram-se diferenças de média de idade (p=0,049) − o Grupo 1 apresentou média de idade menor do que o Grupo 2.

A avaliação do comportamento da proteinúria e creatinina por momentos de avaliação e HAS foi realizada via modelo GEE.

Conforme ilustrado na tabela 1, os Grupos 1 e 2 apresentaram comportamentos similares de proteinúria (p=0,127) e creatinina (p=0,514) ao longo do tempo.

**Tabela 1:** Resultados do modelo equações de estimação generalizadas para proteinúria e creatinina

Variáveis	Coeficiente (IC95%)	Valor de p
Proteinúria, g/24 horas		
HAS*	-1,70 (-4,49-1,09)	0,232
Tempo, pós^†^	-3,28 (-5,68- -0,88)	0,007
Interação tempo *versus* HAS	1,99 (-0,56-4,54)	0,127
Constante	4,35 (1,76-6,94)	0,001
Creatinina, mg/dL		
HAS*	0,09 (-0,49-0,68)	0,757
Tempo^†^	-0,29 (-0,69-0,11)	0,154
Interação tempo *versus* HAS	0,15 (-0,30-0,61)	0,514
Constante	1,10 (0,76-1,44)	<0,001

n=58 observações relativas a 29 pacientes.

* sem HAS como referência; ^†^ tendo como referência os valores pré terapia de indução.

IC95%: intervalo de confiança de 95%; HAS: hipertensão arterial sistêmica.

Além disso, para a proteinúria, observou-se apenas o efeito de tempo (p=0,007), mas não de HAS (p=0,232). Assim, nota-se redução nos níveis da proteinúria (redução de 3,28g/24 horas, em média) do início para o momento pós, como apresentado na tabela 2. Já para a creatinina, não se observaram efeitos de HAS (p=0,757) e nem de tempo (p=0,154). Esses comportamentos médios podem ser visualizados nas figuras 1 e 2.

**Tabela 2:** Medidas-resumo da proteinúria e creatinina por hipertensão arterial sistêmica

Variáveis	Momento de avaliação		
	Pré	Pós 3-6 meses	Variação*
Proteinúria, g/24 horas			
Sem HAS			
Média±DP	4,35±4,13	1,07±1,08	-3,28±3,82
Mediana (Min-Máx)	3,2 (1,00-14,79)	0,37 (0,15-3,22)	-2,26 (-13,18- -0,79)
HAS			
Média±DP	2,65±2,38	1,36±1,73	-1,29±2,01
Mediana (Min-Máx)	2,26 (0,46-9,80)	0,57 (0,12-6,85)	-1,35 (-6,01-3,46)
Creatinina, mg/dL			
Sem HAS			
Média±DP	1,10±0,54	0,81±0,15	-0,29±0,63
Mediana (Min-Máx)	0,90 (0,70-2,30)	0,80 (0,50-1,00)	0,00 (-1,80-0,10)
HAS			
Média±DP	1,19±1,09	1,06±0,64	-0,14±0,51
Mediana (Min-Máx)	0,90 (0,60-5,70)	0,90 (0,60-3,60)	0,00 (-2,10-0,40)

n=9 e n=20, respectivamente para os Grupos 1 e 2.

^*^ valores pós - pré terapia de indução. HAS: hipertensão arterial sistêmica; DP: desvio padrão.

**Figura 1:** Estimativa das médias de creatinina, por momento de avaliação, segundo hipertensão arterial sistêmica HAS: hipertensão arterial sistêmica.

**Figura 2:** Estimativa das médias de proteinúria por momento de avaliação, segundo hipertensão arterial sistêmica HAS: hipertensão arterial sistêmica.

Devido ao fato dos grupos terem apresentado médias distintas de idade, procedeu-se ao ajuste do modelo de regressão com a inclusão da idade no modelo.

Conforme tabela 3, nota-se que a idade mostrou-se significante para a proteinúria (p=0,019), mas não para creatinina (p=0,745). Assim, para a proteinúria, observou-se redução de 0,06g/24 horas a cada aumento de 1 ano de idade. Novamente, pode-se notar que os pacientes com HAS e sem HAS apresentaram comportamentos similares de proteinúria (p=0,055) e creatinina (p=0,478) ao longo do tempo. Além disso, para a proteinúria, novamente observou-se apenas o efeito de tempo (p<0,001), mas não de HAS (p=0,255). Desta forma, nota-se redução nos níveis da proteinúria (redução de 3,28g/24 horas, em média) do início para o momento pós. Já para a creatinina, não se observaram efeitos de HAS (p=0,690) e nem de tempo (p=0,104).

**Tabela 3:** Resultados do modelo equações de estimação generalizadas para proteinúria e creatinina com ajuste pela inclusão da idade ao modelo

Variáveis	Coeficiente (IC95%)	Valor de p
Proteinúria		
HAS*	-1,05 (-2,85-0,76)	0,255
Tempo, pós^†^	-3,28 (-4,97- -1,59)	<0,001
Interação tempo *versus* HAS	1,99 (-0,05-4,02)	0,055
Idade	-0,06 (-0,12--0,01)	0,019
Constante	7,15 (4,40-9,90)	<0,001
Creatinina		
HAS*	0,13 (-0,50-0,76)	0,690
Tempo, pós^†^	-0,29 (-0,64-0,06)	0,104
Interação tempo *versus* HAS	0,15 (-0,27-0,57)	0,478
Idade	0,00 (-0,03-0,02)	0,745
Constante	1,26 (0,20-2,31)	0,020

n=58 observações relativas a 29 pacientes.

* sem HAS como referência; ^†^ tendo como referência os valores pré terapia de indução.

IC95%: intervalo de confiança de 95%; HAS: hipertensão arterial sistêmica.

Verificou-se redução da percentagem de alteração tanto no Grupo 1 (p=0,013) como no Grupo 2 (p=0,001). que pode ser notado na figura 3, a qual demonstra uma alteração variação maior no Grupo 1. Observou-se também, redução relativa de proteinúria em pacientes do Grupo 1 (p<0,001) e do Grupo 2 (p=0,001) representada graficamente na figura 4. Conforme tabela 4, não se verificaram distribuições distintas de alteração de proteinúria por HAS (p=0,689) e nem diferenças de médias de variações relativas de proteinúria por HAS (p=0,091).

**Figura 3:** Proporção de alteração de proteinúria após a intervenção e respectivo intervalo de confiança de 95%, segundo hipertensão arterial sistêmica HAS: hipertensão arterial sistêmica.

**Figura 4:** Média da variação relativa de proteinúria e respectivo intervalo de confiança de 95%, segundo hipertensão arterial sistêmica HAS: hipertensão arterial sistêmica.

**Tabela 4:** Distribuição dos pacientes por classificação de proteinúria pós e variação relativa da proteinúria

Variáveis	Total	Sem HAS	HAS	Valor de p
Proteinúria* pós				0,689
Sem alteração (≤0,5g/24 horas)	13/28 (46,4)	5/9 (55,6)	8/19 (42,1)	
Alterado (>0,5g/24 horas)	15/28 (53,6)	4/9 (44,4)	11/19 (57,9)	
Variação^†^ relativa da proteinúria				0,091^‡^
Média±DP	-52,88±43,32	-73,15±21,76	-43,75±47,79	
Mediana (Min-Máx)	-57,24 (-96,00; 102,06)	-79,00 (-96,00; -38,67)	-52,76 (-92,22; 102,06)	

Resultados expressos por n (%) ou %.

Variação relativa da proteinúria: n=9 e n=20, respectivamente paraos Grupos 1 e 2. Teste de Kolmogorov-Smirnov para normalidade na distribuição da variação relativa da proteinúria (p=0,317).

* somente para os pacientes com alteração de proteinúria na avaliação inicial; ^†^ valores pós - pré terapia de indução. ^‡^ teste exato de Fisher ou *t* de Student.

HAS: hipertensão arterial sistêmica; DP: desvio padrão.

## DISCUSSÃO

A partir da análise dos resultados em questão, levando em conta os dados obtidos no desfecho primário, demonstrou-se similaridade no comportamento da proteinúria entre os pacientes com e sem HAS. Ambos obtiveram redução 3,28g/24 horas em média. Esse resultado sugere que o fato dos pacientes hipertensos já utilizarem um antiproteinúrico e, possivelmente, apresentarem lesões de microvasculatura relacionados à HAS não acarreta efeito direto significativo nesse parâmetro.

Considerando ainda os elementos do desfecho primário, fica evidente que o nível sérico de creatinina, assim como a proteinúria, não apresenta efeito secundário à variável HAS. O que se observa na análise gráfica são níveis séricos mais elevados de creatinina entre os hipertensos, os quais atribuímos aos fatores idade e HAS.

Em nefropatias progressivas, como no caso da hipertensiva, a disfunção grave da barreira capilar glomerular de proteínas circulantes provoca sobrecarga de proteína de células epiteliais tubulares e ativação do complemento intrarrenal, que é responsável pela propagação dos danos para o compartimento túbulo-intersticial.^(18)^

A passagem anormal de proteínas plasmáticas através da parede capilar glomerular é o fator responsável por mais lesões podocitárias e progressão para glomeruloesclerose. Porém devemos destacar outros mecanismos que levam à ativação das células tubulares proximais, inflamação intersticial e fibrose, como a própria toxicidade da albumina, além de transferrina e imunoglobulinas ultrafiltradas, e ativação da via do complemento.^(18)^

Um dos primeiros ensaios clínicos apoiando o conceito de proteinúria como um fator de risco independente para a progressão da doença renal foi a *Modification of Diet in Renal Disease* (MDRD). Desde então, numerosas análises confirmaram essa observação. Assim, medicações hipotensoras para pacientes com doença renal baseiam-se na eficácia desses agentes na redução da proteinúria.^(19)^

Vários estudos que demonstraram renoproteção com a terapia em uso de IECA ou BRA também relataram redução na proteinúria. Uma análise de estudos em pacientes com hipertensão e nefropatia diabética e em pacientes sem diabetes e com hipertensão e nefropatia revelou que as mudanças iniciais na proteinúria mostraram relação favorável com grau de deterioração renal a longo prazo.^(19)^

Os parâmetros renais da proteinúria e creatinina sérica têm sido amplamente aceitos como medidas de resposta de curto prazo, pois são medidas não invasivas, facilmente quantificáveis e podem refletir a lesão renal. Eles são usados individualmente e em combinação nos ensaios clínicos, para determinar a eficácia de novos medicamentos no paciente lúpico, embora sua utilidade na previsão do desfecho renal em longo prazo não tenha sido verificada.^(12)^

Dall’Era et al.,^(11)^ demonstram resultados que fornecem um argumento convincente para o uso do nível absoluto de proteinúria isoladamente como medida de resposta terapêutica e um marcador prognóstico valioso a longo prazo. Porém o nível sérico absoluto de creatinina após 1 ano não adicionou muito ao valor preditivo do nível absoluto de proteinúria isolado. Nesse estudo, também sugerem que o critério frequentemente usado de proteinúria de 0,5g ao dia pode não ser ideal.^(12)^

Um estudo na população brasileira demonstrou que a proteinúria inferior 0,8g/24 horas aos 12 meses de seguimento foi o único melhor preditor de um bom desfecho renal em longo prazo, em um grupo etnicamente diverso de pacientes com nefrite grave. Em relação às classes histológicas, a glomerulonefrite membranosa parece ter curso de longo prazo mais favorável em comparação com as formas proliferativas da doença, e há algumas preocupações de que o desempenho do alvo da proteinúria proposto possa ser diferente em pacientes com formas proliferativas em relação à membranosa.^(20)^

Assim, esperávamos, em nosso estudo, que os pacientes hipertensos, pelo fato de já fazerem uso de um nefroprotetor, apresentassem, em curto prazo, uma resposta mais satisfatória em relação aos não hipertensos, o que não se confirmou.

Durante a análise do desfecho secundário, avaliamos que, apesar dos pacientes hipertensos apresentarem um valor de proteinúria inicial inferior, o decréscimo percentual de proteinúria dos não hipertensos não apresentou alteração estatística comparativa frente aos dois grupos, apesar de redução de 73,15% entre os sem HAS *versus* 43,75% dos com HAS.

Tal diferença entre o decréscimo é esperada, uma vez que os hipertensos que realizam uso prévio de um antiproteinúrico já apresentam valor inicial reduzido em relação aos demais, no entanto outro fator a ser considerado é provável albuminúria residual relacionada à doença hipertensiva e não necessariamente a atividade da doença lúpica.^(21)^

Ainda nesse contexto, no que se refere ao alvo de proteinúria, 55,6% dos não hipertensos atingiram o alvo estabelecido de 0,5g/24 horas frente a 42,1% dos hipertensos. Esses dados demonstram que 13,5% a mais dos não hipertensos submetidos apenas à terapia de indução atingiram o valor-alvo. Essa diferença não foi significativamente considerável em nossa análise, e, nesse resultado, devemos levar em conta a proteinúria secundária à doença hipertensiva de base.

O alvo ideal de proteinúria é discutível, porém é inquestionável a importância de atingi-lo, já que ele se apresenta como marcador de aterosclerose e doença vascular na população geral e em pacientes com DM, com risco estimado de 50% maior de doença aterosclerótica coronariana do que os controles saudáveis.^(22)^

Assim, proteinúria de doença renal lúpica ativa ou DRC no LES está associada a doenças cardiovasculares e, portanto, é considerada fator prognóstico. Em pacientes com LES, há aumento na taxa de mortalidade relacionada à aterosclerose prematura, com maior prevalência de obstrução significativa de artérias coronárias em comparação com os controles saudáveis. Em geral, os pacientes com LES têm risco maior de aterosclerose acelerada do que os pacientes com DM.^(22)^

Tendo como objetivo o aumento de sobrevida de pacientes lúpicos com disfunção renal, o parâmetro da proteinúria se torna tanto valor diagnóstico como prognóstico essencial para o seguimento desses indivíduos. Levando em conta comorbidades associadas a doenças autoimunes, como hipertensão e diabetes, faz-se necessário maior cuidado; possivelmente esses pacientes necessitem de parâmetros de proteinúria individuais para definição de atividade ou remissão da doença.^(22)^

Nosso estudo apresenta uma limitação importante, no que se refere ao tamanho das amostras, além da heterogeneidade elas, deixando a análise estatística prejudicada. Porém, uma vez que se limita a incluir apenas pacientes com biópsia, um número considerável de pacientes foi excluído, dificultando encontrar um número adequado e homogêneo para os grupos.

## CONCLUSÃO

Neste estudo, fica evidente a similaridade no comportamento da proteinúria, após tratamento de indução para nefrite lúpica, considerando o efeito da hipertensão arterial sistêmica. Apesar da diferença de proteinúria inicial entre os grupos, após a terapia não se observou o efeito significativo da hipertensão arterial sistêmica no decréscimo percentual de proteinúria. Portanto, a comorbidade prévia não se mostrou impeditiva para que estes pacientes atingissem a meta de proteinúria preconizada.

A partir dos resultados obtidos, vê-se a necessidade de estudos aprofundados com maior número de pacientes, para não haver maiores interferências referente à heterogeneidade entre as amostras.
